# Mastocytosis presenting with mast cell‐mediator release‐associated symptoms elicited by cyclo oxygenase inhibitors: prevalence, clinical, and laboratory features

**DOI:** 10.1002/clt2.12132

**Published:** 2022-03-16

**Authors:** Tiago Azenha Rama, José Mário Morgado, Ana Henriques, Luis Escribano, Iván Alvarez‐Twose, Laura Sanchez‐Muñoz, André Moreira, José Romão, Alberto Órfão, Almudena Matito

**Affiliations:** ^1^ Serviço de Imunoalergologia Centro Hospitalar Universitário São João Porto Portugal; ^2^ Serviço de Imunologia Departamento de Patologia Faculdade de Medicina da Universidade do Porto Porto Portugal; ^3^ Instituto de Estudios de Mastocitosis de Castilla La Mancha and CIBERONC CB16/12/00400 Hospital Virgen del Valle Toledo Spain; ^4^ Spanish Network on Mastocytosis (REMA) Toledo and Salamanca Spain; ^5^ Servicio General de Citometría Centro de Investigación del Cáncer (IBMCC‐CSIC/USAL and IBSAL) CIBERONC CB16/12/00400 and Departamento de Medicina Universidad de Salamanca Salamanca Spain; ^6^ EPIUnit Instituto de Saúde Pública da Universidade do Porto Porto Portugal; ^7^ Instituto de Ciências Biomédicas de Abel Salazar Porto Portugal; ^8^ Serviço de Anestesiologia Centro Hospitalar do Porto Porto Portugal

**Keywords:** anaphylaxis, mast cell‐mediator release‐associated symptoms, mast cells, mastocytosis, non‐steroidal anti‐inflammatory drug hypersensitivity

## Abstract

**Background:**

Nonsteroidal anti‐inflammatory drugs (NSAIDs) are frequently avoided in mastocytosis, because of a potential increased risk for drug hypersensitivity reactions (DHRs) due to inhibition of cyclo‐oxygenase (COX), subsequent depletion of prostaglandin *E*_2_ and release of leukotrienes.

**Objectives:**

Here, we aimed at determining the prevalence of mast cell (MC) mediator release symptoms triggered by NSAIDs in mastocytosis patients and the associated clinical and laboratory features of the disease.

**Methods:**

Medical records from 418 adults to 223 pediatric mastocytosis patients were retrospectively reviewed. Patients were classified according to tolerance patterns to NSAIDs and other COX inhibitors (COXi) and compared for epidemiological, clinical and laboratory findings.

**Results:**

Overall, 87% of adults and 91% of pediatric patients tolerated NSAIDs and other COXi. Among adult and pediatric patients presenting DHRs, 5% and 0% reacted to multiple NSAIDs, 4% and 0.7% were single reactors, and 3% and 8% were single reactors with known tolerance to paracetamol but unknown tolerance to other COXi, respectively. Among adults, hypersensitivity to ≥2 drugs was more frequent among females (*p* = 0.009), patients with prior history of anaphylaxis to triggers other than NSAIDs or other COXi and *Hymenoptera* venom (*p* = 0.009), presence of baseline flushing (*p* = 0.02), baseline serum tryptase ≥48 ng/ml (*p* = 0.005) and multilineage *KIT* mutation (*p* = 0.02). In contrast, tolerance to NSAIDs and other COXi was more frequent among males (*p* = 0.02), in patients with anaphylaxis caused by *Hymenoptera* venom (*p* = 0.02), among individuals who had skin lesions due to mastocytosis (*p* = 0.01), and in cases that had no baseline pruritus (*p* = 0.006). Based on these parameters, a score model was designed to stratify mastocytosis patients who have never received NSAIDs or other COXi apart from paracetamol, according to their risk of DHR.

**Conclusions:**

Our results suggest that despite the frequency of MC mediator related symptoms elicited by NSAIDs and other COXi apart from paracetamol is increased among mastocytosis patients versus the general population, it is lower than previously estimated and associated with unique disease features. Patients that tolerated NSAIDs and other COXi following disease onset should keep using them. In turn, adults with unknown tolerance to such drugs and a positive score should be challenged with a preferential/selective COX‐2 inhibitor, while the remaining may be challenged with ibuprofen.

**ABBREVIATIONS**
ASA ‐: Acetylsalicylic acid
ASM –: Aggressive systemic mastocytosis
AUC ‐: Area under the curve
BM –: Bone marrow
BMM –: Bone marrow mastocytosis
BMMC –: Bone marrow mast cell
CI ‐: Confidence interval
CM –: Cutaneous mastocytosis
COX –: Cyclooxygenase
COXi –: Cyclooxygenase inhibitors
DCM ‐: Diffuse cutaneous mastocytosis
DHR –: Drug hypersensitivity reactions
GI ‐: Gastrointestinal
HVA –: Hymenoptera venom anaphylaxis
Ig ‐: Immunoglobulin
ISM –: Indolent systemic mastocytosis
LT ‐: Leukotriene
MC –: Mast cell
MCA –: Mast cell activation
MIS –: Mastocytosis in the skin
NPV ‐: Negative predictive value
NSAIDs –: Non‐steroidal anti‐inflammatory drugs
PDGFR ‐: Platelet‐derived growth factor receptor
PG –: Prostaglandin
PPV ‐: Positive predictive value
REMA –: Spanish Network on Mastocytosis
ROC ‐: Receiver operating characteristic
sBT –: serum baseline tryptase
SM‐AHN –: Systemic mastocytosis with an associated hematological neoplasm
SSM –: Smouldering systemic mastocytosis
WDSM –: Well‐differentiated systemic mastocytosis
WHO ‐: World Health Organization

## 1 INTRODUCTION

Mastocytosis encompasses a heterogeneous group of rare diseases characterized by the accumulation of clonal and phenotypically aberrant mast cells (MC) in different tissues and organs, such as the skin, bone marrow (BM) and the gastrointestinal (GI) tract.^1^, ^2^ Mastocytosis might affect children and adults, independently of gender,^1^, ^2^ with an estimated prevalence of ≈9 cases per 100,000 individuals.^3^, ^4^ Currently, the World Health Organization (WHO) defines seven diagnostic subtypes of mastocytosis that include cutaneous mastocytosis (CM), five subtypes of systemic mastocytosis (SM)—that is indolent SM (ISM), smouldering SM (SSM), SM associated with another hematological (non‐MC lineage) neoplasm (SM‐AHN), aggressive SM (ASM), MC leukemia (MCL) ‐ and MC sarcoma.^5^ Moreover, a provisional diagnostic subtype of ISM—that is bone marrow mastocytosis (BMM)—is also defined by WHO,^5^ and another variant of well differentiated SM has been recently characterized.^6^, ^7^ Most mastocytosis patients present with mild to severe symptoms caused by the release of MC mediators, infiltration of tissues by MC, or both,^1^, ^2^ in association with an overall higher prevalence of hereditary alpha tryptasemia.^8^

Activated MC release multiple vasoactive, proinflammatory, chemotactic and immunomodulatory mediators which are both prestored in granules and produced de novo.^9^ A broad variety of triggers for MC activation have been described so far, which, among others, include cyclooxygenase inhibitors (COXi).^10^, ^11^ According to the Anatomical Therapeutical Chemical (ATC)/WHO classification, COXi include traditional nonsteroidal anti‐inflammatory drugs (NSAIDs)—butylpyrazolidines, acetic acid derivatives and related substances, oxicams, propionic acid derivatives, fenamates, and other not otherwise classified drugs (e.g., nimesulide, nabumetone, clonixin lysine) —COX‐2 selective NSAIDs (i.e. coxibs), and analgesics and antipyretics (henceforth other COXi) such as salycilates (e.g. acetylsalicylic acid, ASA), pyrazolones and paracetamol.^12^ NSAIDs and ASA inhibit COX, which results in pain control and both anti‐inflammatory and antipyretic effects. In contrast, paracetamol exerts a limited inhibitory effect on COX‐1, lacks anti‐inflammatory effects and exerts its analgesic and antipyretic effects through central nervous system COX‐2 inhibition.^13^

In Spain, the prevalence of hypersensitivity to NSAID ranges between 1% and 3% in the (adult) general population,^14^ while it is estimated to be 1.2% in children.^15^ In contrast to the general population, previous studies in mastocytosis showed a greater prevalence of NSAID hypersensitivity of up to 14% in adults and 2% in pediatric patients.^16^ In addition, NSAIDs have been reported to cause anaphylaxis in between 2% and 11% of adult mastocytosis patients.^17^, ^18^, ^19^, ^20^, ^21^ Because of this, strict avoidance of NSAIDs in mastocytosis is often recommended in routine clinical practice, due to safety concerns.^22^

From a pathogenic point of view, patients might display NSAID or other COXi hypersensitivity to specific drugs or structurally related groups of drugs—single reactors—and to different structurally unrelated drugs—multiple reactors.^23^ Pyrazolones frequently cause IgE‐mediated hypersensitivity reactions,^24^ while NSAIDs and other COXi most commonly induce IgE‐independent reactions through a COX‐1‐related mechanism.^25^ COX‐1 inhibition depletes protective PGE_2_,^25^, ^26^ resulting in increased production of cysteinyl‐leukotrienes^27^ that leads to symptoms ranging from urticaria to anaphylaxis.^25^

NSAID hypersensitive patients might react to COX‐1 inhibitors while tolerating weak COX‐1 inhibitors (e.g. paracetamol) and COX‐2 (preferential or selective) inhibitors (i.e. meloxicam or coxibs, respectively), that induce a lower decrease in PGE_2_ with lower production of leukotrienes.^28^ Whether the mechanisms involved in NSAID‐associated release of MC mediators in mastocytosis is similar or not to that described for the general population, currently remains unclear.

Here, we retrospectively analyzed the prevalence of MC‐mediator release‐associated symptoms triggered by NSAIDs and other COXi in a large series of mastocytosis patients, and compared the clinical and laboratory features of these patients with those of other mastocytosis patients, in order to search for a potentially unique clinical and laboratory profile associated with hypersensitivity to these drugs.

## 2 MATERIALS AND METHODS

### 2.1 Study design

Randomly selected medical records from a total of 641 patients—418 adults and 223 children and adolescents <18 years old—diagnosed with mastocytosis as per the WHO criteria at the Spanish Network on Mastocytosis (REMA) and that had been followed for a minimum period of 1 year, were retrospectively reviewed. Eighty‐two pediatric patients with insufficient or inconsistent clinical data were subsequently excluded from the analysis. Ninety patients (36 adult and 54 pediatric patients) who had never received NSAIDs or other COXi following the onset of mastocytosis, but that had tolerated paracetamol, were also further excluded from the study. Data recorded on the remaining 469 patients (Figure S1)—382 adults and 87 children and adolescents—included: diagnostic subtypes of mastocytosis, basal MC mediator‐related symptoms (pruritus, flushing and GI symptoms) and prior history of anaphylaxis and its respective triggers, among other clinical and laboratory features of the disease, which are described in more detail in Table 1.

**TABLE 1:** Demographic, clinical and laboratory features of adult and pediatric mastocytosis patients included in the study (*n* = 469)

		Adults (*n* = 382)	Children and adolescents (*n* = 87)
**Sex (female)**		204 (53%)	38 (43%)
**Age (years)**		48 (19‐85)	10 (2‐17)
**Age at onset of mastocytosis (years)**		33 (0‐82)	4 (0‐10)
**Diagnosis**	CM	22 (6%)	86 (99%)
	MIS	24 (6%)	0 (0%)
	ISM	197 (52%)	1 (1%)
	BMM	115 (30%)	0 (0%)
	SSM	4 (1%)	0 (0%)
	ASM	10 (3%)	0 (0%)
	ISM‐AHN	7 (2%)	0 (0%)
	MCL	3 (1%)	0 (0%)
	WDSM	17 (5%)	1 (1%)
**Clinical signs and symptoms of mastocytosis**	Flushing	202 (53%)	30 (34%)
	Pruritus	160 (42%)	48 (55%)
	GI symptoms	185 (48%)	25 (29%)
	Skin lesions	257 (67%)	87 (100%)
	Anaphylaxis:	174 (46%)	4 (5%)
	*HVA*	70 (40%)	0 (0%)
	*Drug allergy*	49 (28%)	3 (75%)
	*Idiopathic*	22 (13%)	1 (25%)
	*Mixed causes*	16 (9%)	0 (0%)
	*Food allergy*	16 (9%)	0 (0%)
	*Other insects*	1 (1%)	0 (0%)
**Allergic sensitization**		151 (40%)	29 (33%)
**Allergic diseases**	Rhinoconjunctivitis	41 (11%)	21 (24%)
	Asthma	21 (6%)	10 (11%)
	Food allergy	52 (16%)	7 (8%)
	Atopic dermatitis	4 (1%)	11 (13%)
**Laboratory findings**	IgE (kU/L)^a^	20.55 (1‐2425)	36.9 (2‐669)
	Eosinophils (x10^9^/L)^b^	0.2 (0.02‐7.8)	0.24 (0.054‐5.98)
	sBT (ng/mL)	24.9 (3.2‐2222)	5.8 (1.1‐149)
**Bone marrow findings**	Major Criterion^c^	189 (57%)	1 (50%)
	% BM MC^c^	0.09 (0‐26)	0.06 (0.04‐0.08)
	KIT mutation:^d^	345 (96%)	0 (0%)
	*D816V*	332 (92%)	0 (0%)
	*D816Y*	4 (1%)	0 (0%)
	*D816H*	4 (1%)	0 (0%)
	*K509I*	3 (1%)	0 (0%)
	*D816A*	1 (0.3%)	0 (0%)
	*815‐816 insertion*	1 (0.3%)	0 (0%)
	Multilineage *KIT* mutation^d^	69 (18%)	1 (6%)
**Imaging findings**	Diffuse osteosclerosis^e^	25 (7%)	‐
**Follow‐up (years)**		14 (1‐65)	10 (3‐17)

*Note*: Results expressed as number of patients and percentage between brackets (rounded to units) or as median and range between brackets.

Abbreviations: ASM, aggressive systemic mastocytosis; BM, bone marrow; BMM, bone marrow mastocytosis; CM, cutaneous mastocytosis; HS, hypersensitivity; HVA, Hymenoptera venom anaphylaxis; ISM, indolent systemic mastocytosis; ISM‐AHN, indolent systemic mastocytosis with an associated hematological neoplasm MC, mast cells; MCL, mast cell leukemia; MIS, mastocytosis in the skin; NS, not statistically significant; sBT, serum baseline tryptase; SSM, smouldering systemic mastocytosis; WDSM, well‐differentiated systemic mastocytosis.

^a^ Analyzed in 339 adults and 62 pediatric patients.

^b^ Studied in 336 adults and 75 pediatric patients.

^c^ Assessed by flow cytometry in 339 adults and 2 pediatric patients.

^d^ Analyzed in 361 adults and 2 pediatric patients.

^e^ Studied in 344 adult patients, not applicable to children/adolescents.

This study was approved by the Ethics Committee of the *Complejo Hospitalario de Toledo* (Toledo, Spain) and every procedure was in accordance with the Declaration of Helsinki. Written informed consent was given by each patient and/or patient legal guardian for collection of clinical data and, in a subgroup of patients also for undergoing drug challenge testing.

### 2.2 Definitions, diagnostic procedures and laboratory tests

Onset of mastocytosis was defined either as the date of first appearance of cutaneous lesions, or in cases who presented in the absence of the typical skin lesions of mastocytosis as the first episode of anaphylaxis, or detection of B and/or C findings.^29^ Diagnosis of mastocytosis was retrospectively revised based on well‐established morphological,^30^ histopathological, immunohistochemical,^31^ immunophenotypic^31^ and molecular criteria,^32^ according to the WHO classification criteria^5^ and more recent criteria for WDSM.^6^, ^7^ Multilineage *KITD816V* mutation was defined as involvement of fluorescence activated cell sorting (FACS)‐purified non‐MC myeloid and/or lymphoid cell populations by this *KIT* mutation as assessed by a previously described^32^ peptide nucleic acid (PNA) polymerase chain reaction (PCR) clamping (PNA‐PCR) technique. In turn, patients with the *KITD816 V* mutation restricted to BM MC were categorized as carrying a MC‐restricted *KITD816V* mutation in BM.^32^ Diagnosis of anaphylaxis followed the 2011 World Allergy Organization Guidelines.^33^ Patients over 18 years old (adults) with cutaneous involvement in the absence of a BM study were categorized as mastocytosis in the skin (MIS), since SM could not be confirmed or ruled out.^34^

Blood tests performed at diagnosis and at follow‐up included: complete blood cell count and differential, routine biochemistry, serum baseline tryptase (sBT; ImmunoCAP Tryptase, Phadia/Thermo Fisher Scientific Inc, Uppsala, Sweden) and both total and specific serum IgE levels (ImmunoCAP total IgE, Phadia/Thermo Fisher Scientific Inc.). Specific IgE levels were measured whenever appropriate (ImmunoCAP allergen components, Phadia/Thermo Fisher Scientific Inc.). In addition, skin tests (e.g. skin prick and intradermal tests) were performed with specific triggers (e.g. *Hymenoptera* venom, aeroallergens, foods and drugs). Allergic sensitization was defined based on positive specific IgE antibodies or a positive skin test.^31^

### 2.3 Diagnosis and classification of hypersensitivity reactions to NSAID or other COXi

Data on MC activation‐associated symptoms induced by NSAIDs and other COXi was retrospectively recorded for each individual patient, through specific anamnesis and review of medical records and it included: the age at disease onset, the type and severity of symptoms and (later) tolerance to each drug used by individual patients, and diagnostic procedures, as follows. Skin tests were performed whenever an underlying IgE‐mediated hypersensitivity (i.e., single reaction to metamizole performed and positive in two adult patients) was suspected. Drug challenge tests were performed using the suspicious drug or a preferential/selective COX‐2 inhibitor, following risk/benefit assessment. Specifically, the suspicious drug (for children) was used when true reactions were unlikely and when the suspicious reaction was mild. Meloxicam or coxibs (for adults) were used when true reactions were likely, if the reaction had been moderate/severe, and when NSAIDs or other COXi were not tolerated following the index episode, as previously recommended for the general population.^35^

Patients who had no MC mediator release‐related symptoms caused by the administration of NSAIDs and other COXi were classified as tolerant. Multiple reactors were defined by the presence of MC activation (MCA)‐associated symptoms caused by ≥2 structurally unrelated NSAIDs or COXi. Those patients who presented reactions elicited by a single NSAID or other COXi were subclassified either as single reactors (those who avoided only that particular drug or group of structurally related drugs while tolerating other NSAIDs after that episode) or as single reactors with known tolerance to paracetamol but unknown tolerance to NSAID and other COXi (those who avoided all NSAIDs and other COXi apart from paracetamol from that moment on).

### 2.4 Adult cohort

Overall, a total of 382 adult mastocytosis patients were studied, from whom 204 (53%) were females and 178 (47%) were males. Median age at study inclusion was of 48 years (range: 19–85 years), and of 33 years (range: 0–82 years) at disease onset. Further details on patient demographics and clinical and laboratory characteristics are featured in Table 1 and causes for anaphylaxis according to the diagnostic subtype of mastocytosis are shown in Table S1.

### 2.5 Pediatric patient series

From 87 pediatric patients analyzed, 38 (44%) were girls, with a median age at the moment of entering the study of 10 years (range: 2–17 years) and at disease onset of 4 months (range: birth—10 years). Further details on patient demographics and clinical and laboratory characteristics are shown in Table 1 and causes for anaphylaxis according to the diagnostic subtype of mastocytosis are displayed in Table S1.

### 2.6 Statistical analyses

For all continuous variables median and range values were calculated, while frequencies were determined for categorical parameters. The Kruskall–Wallis or Mann–Whitney *U* tests, and the *χ* ^2^ or Fisher's exact tests were used to assess the statistical significance of differences observed between (2 or ≥2) groups, for continuous and categorical variables, respectively. Receiver operating characteristic (ROC) curve analysis was used to define optimal cut‐off values to predict for hypersensitivity to NSAIDs or other COXi. In order to identify the best combination of independent factors associated with hypersensitivity to multiple NSAIDs, multivariate binary logistic regression analysis was used. Only variables that showed statistically significant differences in the univariate study were selected for the multivariate analysis. Two models were built: the first model focused on identifying individuals with hypersensitivity to ≥2 NSAIDs or other COXi versus those that were tolerant to these drugs, while the second model focused on the discrimination of patients that were tolerant versus hypersensitive patients to ≥1 drugs. ROC curves were obtained, and the area under the curve (AUC) was used in order to assess the best combination of independent factors for models 1 and 2. For all statistical analyses the SPSS® for Windows (version 23.0; IBM Corporation) and RStudio (version 1.3.959) software programs were used. *p*‐Values <0.05 were considered to be associated with statistical significance.

## 3 RESULTS

Overall, 411/469 (88%) patients were tolerant, while 20 (4%) were multiple reactors, 24 (5%) single reactors and 14 (3%) single reactors with known tolerance to paracetamol and unknown tolerance to NSAIDs and other COXi apart from paracetamol.

### 3.1 Adult cohort

Most adult mastocytosis patients (332/382 cases; 87%) tolerated NSAIDs. In contrast, hypersensitivity reactions were observed in 50 (13%) cases (Table 2), corresponding to 20 (5%) multiple reactors (all but four of them tolerated paracetamol), 17 (4%) single reactors and 13 (3%) single reactors with known tolerance to paracetamol and unknown to other COXi (Table 2). Noteworthy, NSAID DHR was the cause for referral/suspicion of mastocytosis in 5 (10%) patients, of which two‐fifths were multiple reactors and three‐fifths were single reactors, and of which four‐fifths had BMM with anaphylaxis, or severe angioedema upon receiving NSAIDs, and one‐fifth had ISM with anaphylaxis upon receiving NSAIDs. Drug challenges were performed in 48/51 patients who reported NSAID DHR, with coxibs (celecoxib in 46 patients and etoricoxib in 2), while 10 and 2 were further challenged with meloxicam and paracetamol, respectively. Three NSAIDs hypersensitive patients had a positive challenge, two with etoricoxib and one with celecoxib, two of which did not tolerate paracetamol. Interestingly, patients presenting with DHR to NSAIDs displayed unique clinical and laboratory features, which are detailed in Table 2.

**TABLE 2:** Demographic, clinical and laboratory features of adult mastocytosis patients distributed according to their pattern of tolerance to NSAIDs and other COX inhibitors (*n* = 382)

		NSAID tolerants (*n* = 332)	HS to NSAIDs (*n* = 50)	*p* Value	Single reactors (*n* = 17)	Single reactors with known tolerance paracetamol and unknown to other COXi (*n* = 13)	Multiple reactors (*n* = 20)	*p* Value
Sex (female)		167 (50%)	37 (74%)	0.002	10 (59%)	11 (85%)	16 (80%)	NS
Age (years)		48 (19–85)	53 (31–81)	0.03	53 (32–70)	54 (32–67)	52 (31–81)	NS
Age at onset of mastocytosis (years)		33 (0–82)	32 (0–72)	NS	35 (0–67)	31 (1–59)	32 (8–72)	NS
Diagnosis	CM	17 (5%)	5 (10%)	NS	2 (12%)	2 (15%)	1 (5%)	NS
	MIS	23 (7%)	1 (2%)	NS	1 (6%)	0 (0%)	0 (0%)	NS
	ISM	171 (52%)	26 (51%)	NS	6 (35%)	8 (62%)	12 (57%)	NS
	BMM	101 (31%)	14 (27%)	NS	7 (41%)	2 (15%)	5 (24%)	NS
	SSM	3 (1%)	1 (2%)	NS	0 (0%)	1 (8%)	0 (0%)	NS
	ASM	6 (2%)	4 (8%)	0.03	1 (6%)	0 (0%)	3 (14%)	NS
	ISM‐AHN	7 (2%)	0 (0%)	NS	0 (0%)	0 (0%)	0 (0%)	NS
	MCL	3 (1%)	0 (0%)	NS	0 (0%)	0 (0%)	0 (0%)	NS
Clinical signs and symptoms of mastocytosis	Flushing	163 (49%)	39 (78%)	<0.001	13 (76%)	10 (77%)	16 (80%)	NS
	Pruritus	129 (39%)	31 (62%)	0.002	7 (41%)	9 (69%)	15 (75%)	NS
	GI symptoms	158 (48%)	27 (54%)	NS	8 (47%)	9 (69%)	10 (50%)	NS
	Skin lesions	220 (67%)	37 (73%)	NS	10 (59%)	11 (85%)	16 (77%)	NS
	Anaphylaxis	136 (41%)	38 (78%)	<0.001	11 (65%)	10 (83%)	17 (85%)	NS
Allergic sensitization		135 (41%)	15 (30%)	NS	8 (47%)	5 (38%)	2 (10%)	0.04
Allergic diseases	Rhinoconjunctivitis	37 (11%)	2 (4%)	NS	1 (6%)	1 (8%)	0 (0%)	NS
	Asthma	16 (5%)	4 (8%)	NS	1 (6%)	3 (23%)	0 (0%)	0.05
	Food allergy	44 (13%)	6 (12%)	NS	3 (18%)	1 (8%)	2 (10%)	NS
	Atopic dermatitis	4 (1%)	0 (0%)	NS	0 (0%)	0 (0%)	0 (0%)	‐
	HVA	73 (22%)	2 (4%)	0.003	0 (0%)	2 (15%)	0 (0%)	0.05
Laboratory findings	IgE (kU/L)^a^	22.1 (1–2425)	16 (2–1062)	NS	37.4 (4.18–1062)	15.65 (2–393)	13 (2–43)	0.06
	Eosinophils (×10^9^/L)^b^	0.2 (0.02–7.8)	0.2 (0.03–1.1)	NS	0.2 (0.05–1.1)	0.3 (0.03–0.45)	0.166 (0.03–0.4)	NS
	sBT (ng/ml)	24 (3.2–1700)	48 (4.4–2222)	0.002	26.5 (4.4–2222)	35.7 (8.12–164)	98.65 (4.92–877)	0.04
	sBT ≥48 ng/ml	89 (27%)	25 (50%)	0.001	4 (24%)	6 (46%)	15 (75%)	0.007
Bone marrow findings	% BM MC^c^	0.09 (0–26)	0.12 (0.0016–23)	NS	0.14 (0.01–7.5)	0.05 (0.0055–0.4)	0.35 (0.0016–23)	0.04
	% BM MC > 0.12%	111 (38%)	23 (50%)	NS	8 (53%)	2 (17%)	13 (68%)	0.02
	Multilineage *KIT* mutation^d^	55 (18%)	14 (28%)	0.006	1 (6%)	3 (23%)	10 (50%)	0.06
Imaging findings	Diffuse osteosclerosis^e^	17 (6%)	8 (17%)	NS	2 (13%)	0 (0%)	6 (32%)	0.01
Follow‐up (years)		13 (1–65)	16.5 (3–43)	0.11	NS	24 (7–40)	17.5 (6–36)	NS

*Note*: Results expressed as number of patients and percentage between brackets (rounded to units) or as median and range between brackets.

Abbreviations: ASM, aggressive systemic mastocytosis; BM, bone marrow; BMM, bone marrow mastocytosis; CM, cutaneous mastocytosis; HS, hypersensitivity; HVA, *Hymenoptera* venom anaphylaxis; ISM, indolent systemic mastocytosis; ISM‐AHN, indolent systemic mastocytosis with an associated hematological neoplasm MC, mast cells; MCL, mast cell leukemia; MIS, mastocytosis in the skin; NS, not statistically significant; sBT, serum baseline tryptase; SSM, smouldering systemic mastocytosis; WDSM, well‐differentiated systemic mastocytosis.

^a^ Analyzed in 339 patients.

^b^ Studied in 336 patients.

^c^ Assessed by flow cytometry in 339 patients.

^d^ Analyzed in 361 patients.

^e^ Studied in 344 patients.

Globally, ASA (19/113 cases; 17%), followed by metamizole and other pyrazolones (21/182 cases; 12%), and coxibs (4/36 cases; 12%, as assessed by drug challenge tests in three‐fourths cases), were those drugs that most frequently elicited DHRs among adult mastocytosis patients, while lower frequencies were found for ibuprofen (20/303 cases; 7%), diclofenac (8/117 cases; 7%), dexketoprofen (2/25 cases; 8%), naproxen (1/29%; 3%), and other less used NSAIDs and other COXi (except for clonixin which showed hypersensitivity in 2/2 multiple reactor patients that received the drug; Table 3). Among multiple reactors, one patient reacted to five different NSAIDs or other COXi (including 1 coxib) while tolerating paracetamol, four patients reacted to 4 NSAIDs or other COXi (including coxibs and paracetamol in three cases, while one case never used paracetamol) and two patients reacted to three NSAIDs or other COXi (paracetamol was involved in one of them, while the other patient tolerated this drug). The remaining 13 cases reacted to two NSAIDs, and all tolerated paracetamol at a dose of 1 g. Three out of four patients who presented with MC mediator‐related symptoms induced by paracetamol were females, and all four had prior history of anaphylaxis not triggered by NSAIDs or other COXi.

**TABLE 3:** NSAIDs and other COX inhibitors as elicitors of MC mediator‐related symptoms in adult mastocytosis patients grouped by type of hypersensitivity

Drug/group	Reaction	Single reactors (*n* = 17)	Single reactors with known tolerance paracetamol and unknown to other COXi (*n* = 13)	Multiple reactors (*n* = 20)	*p* Value	Total
Ibuprofen	Total	2/9 (22%)	5/5 (100%)	13/14 (93%)	<0.001	20/303 (7%)
	Anaphylaxis	0/0 (0%)	2/5 (40%)	9/14 (64%)	0.002	11/303 (4%)
ASA	Total	3/5 (60%)	5/7 (71%)	11/12 (92%)	NS	19/113 (17%)
	Anaphylaxis	0/0 (0%)	5/7 (71%)	9/12 (75%)	0.008	14/113 (12%)
Metamizole and other pyrazolones	Total	10/14 (71%)	1/1 (100%)	10/11 (91%)	NS	21/182 (12%)
	Anaphylaxis	5/14 (36%)	1/1 (100%)	5/11 (50%)	NS	11/182 (6%)
Diclofenac	Total	3/6 (50%)	0/0 (0%)	5/6 (83%)	NS	8/117 (7%)
	Anaphylaxis	3/6 (50%)	0/0 (0%)	4/6 (66%)	NS	7/117 (6%)
Coxibs	Total	0/4 (0%)	0/5 (0%)	4/14 (29%)	NS	4/36 (11%)
	Anaphylaxis	0/0 (0%)	0/0 (0%)	2/14 (14%)	NS	2/36 (6%)
Paracetamol	Total	0/17 (0%)	0/13 (0%)	4/20 (29%)	0.03	4/380 (1%)
	Anaphylaxis	0/0 (0%)	0/0 (0%)	3/20 (15%)	0.03	3/380 (1%)
Clonixin	Total	0/0 (0%)	0/0 (0%)	2/2 (100%)	–	2/2 (100%)
	Anaphylaxis	0/0 (0%)	0/0 (0%)	1/2 (50%)	–	1/2 (50%)
Dexketoprofen	Total	0/3 (0%)	0/0 (0%)	2/2 (100%)	NS	2/25 (8%)
	Anaphylaxis	0/0 (0%)	0/0 (0%)	0/0 (0%)	NS	0/0 (0%)
Nabumetone	Total	0/1 (0%)	0/0 (0%)	1/1 (100%)	NS	1/2 (50%)
	Anaphylaxis	0/0 (0%)	0/0 (0%)	0/0 (0%)	–	0/0 (0%)
Naproxen	Total	0/2 (0%)	0/0 (0%)	1/1 (100%)	NS	1/29 (3%)
	Anaphylaxis	0/0 (0%)	0/0 (0%)	1/1 (100%)	NS	1/29 (3%)
Aceclofenac	Total	0/0 (0%)	1/1 (100%)	1/1 (100%)	NS	2/16 (13%)
	Anaphylaxis	0/0 (0%)	0/0 (0%)	1/1 (100%)	–	1/16 (6%)
Ketorolac	Total	0/1 (0%)	0/0 (0%)	0/0 (0%)	–	0/5 (0%)
	Anaphylaxis	0/0 (0%)	0/0 (0%)	0/0 (0%)	–	0/0 (0%)
Meloxicam	Total	0/3 (0%)	0/4 (0%)	0/6 (0%)	–	0/22 (0%)
	Anaphylaxis	0/0 (0%)	0/0 (0%)	0/0 (0%)	–	0/0 (0%)

*Note*: Results expressed as number of patients who had reactions (total), and of patients who had anaphylaxis out of all patients in the group who used the drug in percentage between brackets (rounded to units). Drugs were classified according to the ATC/WHO classification system as non‐steroidal anti‐inflammatory drugs (NSAIDs): aceclofenac, coxibs, dexketoprofen, diclofenac, ibuprofen, ketorolac, meloxicam, naproxen; or other COX inhibitors: acetylsalicylic acid, clonixin, metamizole and other, nabumetone, pyrazolones, paracetamol.

Abbreviation: ASA, acetylsalicylic acid.

Concerning the specific symptoms presented during DHRs, 15 (30%) cases only had mucocutaneous manifestations (urticaria, pruritus, flushing and/or angioedema), 33 (66%) developed anaphylaxis, 1 (2%) had (reproducible) nasal symptoms (rhinorrhea and sneezing) with ASA and ibuprofen, and 1 (2%) had (reproducible) emesis and abdominal cramping with metamizole and ibuprofen (Table 4), while none had isolated bronchospasm. Upon comparing the clinical and laboratory features of mastocytosis patients presenting with mucocutaneous manifestations versus those with anaphylaxis, the later were more prone to display multilineage involvement of BM by the *KITD816 V* mutation—13/33 (39%) versus 1/15 (7%), *p* = 0.03—with a trend for a higher frequency of patients with sBT≥48 ng/ml—20 (61%) versus 5 (33%), *p* = 0.08. In turn, those that only showed mucocutaneous manifestations were more prone to mastocytosis‐associated skin lesions—14 (93%) versus 21 (64%), *p* = 0.05. No significant differences were found among cases presenting with mucocutaneous manifestations versus anaphylaxis as regards the diagnostic subtype of mastocytosis, age at disease onset, duration of disease, gender, allergic sensitization, type of allergic disease, baseline manifestations, BMMC burden, total serum IgE levels, presence of diffuse osteosclerosis, absolute eosinophil blood count and type of tolerance. During DHRs, multiple reactors less frequently had presyncope (*p* = 0.05), once compared to the two groups of single reactor patients (Table 4). Overall, anaphylaxis caused by NSAIDs or other COXi occurred in 33/382 (9%) patients who had used these drugs, and this accounted for 16% of adult mastocytosis cases who previously had presented with anaphylaxis unrelated to NSAIDs or other COXi. The most frequent culprit for anaphylaxis was ASA (14/113 cases; 12%), followed by pyrazolones (11/182 cases; 6%) and diclofenac (7/11 cases; 6%; Table 3). Of note, reactions caused by coxibs, of which three‐fourths occurred during drug challenges, consisted of anaphylaxis in two cases, angioedema in one case and urticaria in the remaining case.

**TABLE 4:** Clinical findings during reactions to NSAIDs and other COX inhibitors, in single versus multiple reactor adult mastocytosis patients

	Single reactors (*n* = 17)	Single reactors with known tolerance paracetamol and unknown to other COXi (*n* = 13)	Multiple reactors (*n* = 20)	*p* Value
Anaphylactic reactions	8 (47%)	11 (85%)	14 (70%)	NS
Pruritus	2 (25%)	0 (0%)	4 (29%)	NS
Hives	1 (13%)	3 (27%)	2 (14%)	NS
Angioedema	1 (13%)	2 (18%)	6 (43%)	NS
Conjunctivitis	0 (0%)	1 (9%)	2 (14%)	NS
Rhinitis	0 (0%)	4 (36%)	4 (29%)	NS
Wheezing	1 (13%)	1 (9%)	1 (7%)	NS
Dyspnea	4 (50%)	2 (18%)	8 (57%)	NS
Abdominal cramping	0 (0%)	1 (9%)	1 (7%)	NS
Diarrhea	0 (0%)	3 (27%)	2 (14%)	NS
Flushing	3 (38%)	3 (27%)	9 (64%)	NS
Presyncope	6 (75%)	4 (36%)	5 (38%)	NS
Syncope	4 (50%)	4 (36%)	7 (50%)	NS
Non‐anaphylactic reactions	8 (53%)	2 (15%)	6 (30%)	NS
Pruritus	5 (63%)	0 (0%)	3 (50%)	NS
Hives	5 (63%)	1 (50%)	3 (50%)	NS
Angioedema	2 (25%)	1 (50%)	0 (0%)	NS
Conjunctivitis	0 (0%)	0 (0%)	0 (0%)	NS
Rhinitis	0 (0%)	1 (50%)	0 (0%)	NS
Wheezing	0 (0%)	0 (0%)	0 (0%)	NS
Dyspnea	0 (0%)	0 (0%)	0 (0%)	NS
Abdominal cramping	0 (0%)	0 (0%)	0 (0%)	NS
Diarrhea	0 (0%)	0 (0%)	0 (0%)	NS
Flushing	5 (63%)	0 (0%)	3 (50%)	NS
Presyncope	0 (0%)	0 (0%)	1 (17%)	NS

*Note:* Results expressed as number of patients and percentage between brackets (rounded to units).

ROC curve analysis showed that sBT levels ≥48 ng/ml and a BMMC burden by flow cytometry ≥0.12% were the best cut‐offs values for predicting reactions to multiple drugs in adult mastocytosis patients, with a sensitivity of 75% and 68%, and a specificity of 78% and 62%, respectively. Interestingly, within the multiple reactor patient group, both the presence of sBT levels ≥48 ng/ml and a BMMC burden ≥0.12% were associated with the presence of multilineage *KIT* mutation (*p* = 0.001 and *p* = 0.002, respectively) and diffuse osteosclerosis (*p* < 0.001 and *p* = 0.002, respectively).

**FIGURE 1:**
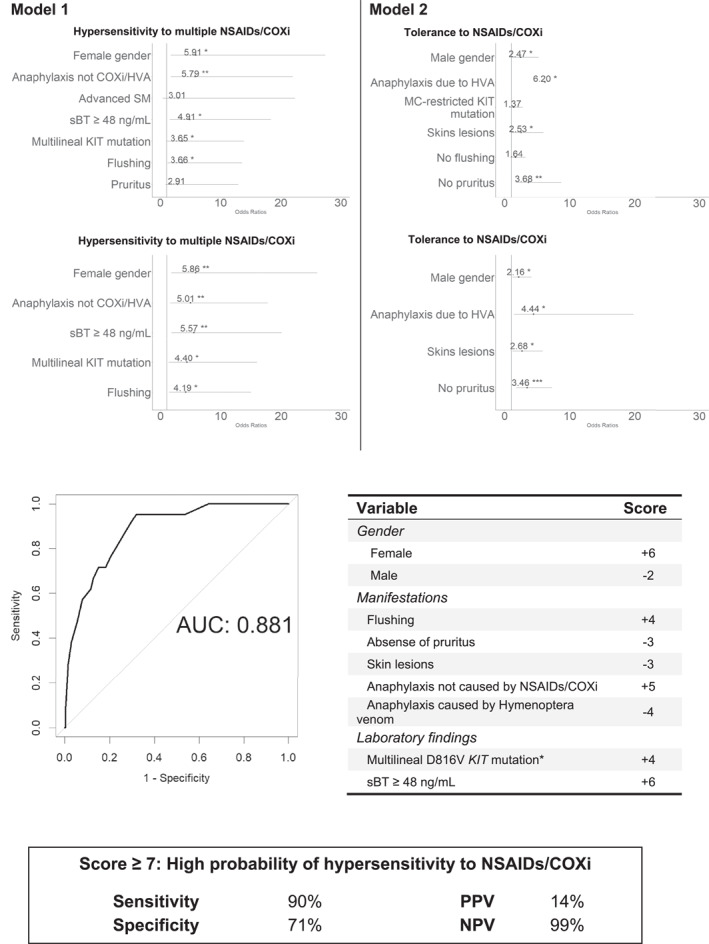
Score model proposed to screen for hypersensitivity to multiple non‐steroidal anti‐inflammatory drugs or other cyclo‐oxygenase inhibitors drugs, in adult patients with mastocytosis

Multivariate analysis showed the following combination of variables to be independent predictors for multiple reactor hypersensitivity: female gender (odds ratio [OR]: 5.9, *p* = 0.009), multilineage involvement of BM cells by the *KIT* mutation (OR: 4.4, *p* = 0.02), past history of anaphylaxis caused by neither NSAIDs or other COXi nor *Hymenoptera* venom (OR: 5.0, *p* = 0.009), sBT ≥48 ng/ml (OR: 5.6, *p* = 0.005) and presence of flushing as a basal MC mediator release related symptom (OR: 4.2, *p* = 0.02). In turn, in a second model built to predict for being tolerant to NSAIDs and other COXi apart from paracetamol, the following independent variables were selected: male gender (OR: 2.2, *p* = 0.02), past history of anaphylaxis caused by *Hymenoptera* venom (OR: 4.4, *p* = 0.02), presence of mastocytosis associated skin lesions (OR: 2.7, *p* = 0.01) and absence of pruritus as a basal MC mediator release related symptom (OR: 3.5, *p* = 0.006). Based on the predictive value of the variables included in both models, a combined score model was built to screen for patients with an increased risk for hypersensitivity to ≥2 NSAIDs or other COXi, with an overall sensitivity of 90% (95% confidence interval [CI]: 70%–99%), a specificity of 71% (95% CI: 67%–75%), and a negative predictive value of 99% (95% CI: 98%–100%) at the expense of a more limited positive predictive value of 14% (95% CI, 12%–16%; Figure 1). Other score models showing a lower area under the curve (AUC) are displayed in Figure S2.

### 3.2 Pediatric cohort

A total of 85 (98%) pediatric patients received ibuprofen (among them, 9 and 4 also received metamizole and ASA, respectively) and 2 (2%) only received metamizole. Overall, 79/87 (91%) patients tolerated NSAIDs and all pediatric patients in this cohort tolerated paracetamol. Drug challenge tests were performed in 25 children/adolescents ‐ ibuprofen in 21 cases, metamizole in 2 cases and celecoxib in the other 2 (14 years and 17 years old) patients—all of whom tested negative. Seven patients (8%) were single reactors with known tolerance to paracetamol and unknown to other COXi—all reactions were elicited by ibuprofen—while the remaining case (0.7%) was a single reactor to metamizole, who tolerated ibuprofen. As opposed to the adults, multilineage *KIT* mutation, diffuse osteosclerosis and BMMC counts were not considered in the study of pediatric patients due to the limited number of pediatric cases who had undergone BM investigations and imaging studies at the moment of closing this study. For all other investigated demographic, clinical and laboratory characteristics, no statistically significant differences were found among pediatric patients that were tolerant versus reactive to NSAIDS and other COXi (Table S2).

Anaphylaxis caused by NSAIDs or other COXi occurred in 2/8 (25%) patients who had a DHR, and it was caused by ibuprofen and metamizole, respectively. Urticaria was the most frequent symptom presented during DHRs in 4/8 (50%) patients (Table S2).

## 4 DISCUSSION

Prescription of NSAIDs and COXi other than paracetamol, is often empirically avoided in mastocytosis patients, because of a general concern for a greater frequency of MCA symptoms triggered by these drugs, particularly anaphylaxis.^11^ However, data about the true prevalence of MCA induced by NSAIDs and other COXi, largely varies in the literature^19^, ^20^, ^21^ and is usually based on limited series of patients with mastocytosis. In this study, based on a large retrospective series of mastocytosis patients we confirmed that a significant fraction (13%) of adults diagnosed with mastocytosis who had previously used these drugs did actually experienced DHRs after using them, less than half of them being reactors to multiple NSAIDs or other COXi. In pediatric patients, the data collected here are less informative, because the sample size and NSAID use were relatively limited. Overall, these results confirm the greater frequency of hypersensitivity reactions to NSAIDs and other COXi in adult mastocytosis compared to the general population in Spain, although the rates might vary depending on the studied patient cohort, as well as potential regional differences.^19^, ^20^, ^21^ Interestingly, the frequency of DHRs to NSAIDs and other COXI found in our adult mastocytosis patients is slightly higher than that previously reported for asthma, but lower than that observed in chronic urticaria.^23^

Despite all the above, DHRs to NSAIDs and other COXi still accounted for a significant fraction of anaphylaxis among adult SM patients with no skin lesions of mastocytosis. Because of this, we subsequently investigated whether patients presenting with DHRs to NSAIDs or other COXi, particularly those that reacted against two or more drugs, had unique clinical and laboratory features that could be used in practice to screen for patients at risk of being multiple reactors to these drugs. In this regard, our findings revealed that adult mastocytosis patients who suffered from DHRs to NSAIDs or other COXi showed a clear female predominance, which is consistent with previous data reported of a higher risk for DHRs among females^36^ and more advanced age, when compared with tolerant patients. In addition, they more frequently had previous history of anaphylaxis related to non‐COXi‐related triggers, other than *Hymenoptera* venom anaphylaxis (HVA), at the same time they more frequently showed angioedema and pruritus, together with signs of more advanced disease including higher sBT levels, together with a greater frequency of multilineage involvement of BM hematopoiesis by the *KIT* mutation, particularly among multiple reactors. Interestingly, these patients showed opposite features from those with HVA, which were mainly male, less symptomatic, had lower sBT levels, absence of multilineage KIT mutation, and a low BMMC burden, as previously described.^37^, ^38^, ^39^.

Overall, this unique clinical and laboratory profile of mastocytosis patients who displayed DHRs to NSAIDs or other COXi (and typically to ≥2 drugs), contrasts, at least in part, to what has been previously reported by others, both for the general population^40^ and different cohorts of mastocytosis patients,^19^, ^20^ which show no clear association between hypersensitivity reactions to NSAIDs and increased sBT or lower sIgE levels. However, it should be noted that such findings were frequently obtained in smaller patient cohorts, relatively enriched in subtypes of SM with lower MC burden (e.g., BMM), in the absence of a clearcut distinction between single and multiple reactors. In fact, both our data and previous studies^19^, ^20^ show that sBT among single reactors is similar to that found in tolerant patients. Compared to our cohort, such studies are based on cohorts with a lower percentage of SM patients presenting with skin involvement, which showed here a higher prevalence of NSAID or other COXi DHR. In this regard, our data confirm other previous observations in mastocytosis which showed a greater frequency of anaphylaxis among patients presenting with greater sBT levels,^18^ even though a very significant proportion of our BMM patients that presented with anaphylaxis showed low sBT. In addition, our results also point out for the first time a potential association between DHRs to two or more NSAIDs or other COXi and a higher MC burden, supporting a role for clonal MC (and potentially also other immune cells) in mastocytosis in favoring hypersensitivity reactions to these drugs. In line with this, patients who were hypersensitive, particularly those that reacted against ≥2 NSAIDs or other COXi, displayed a higher frequency of multilineage *KIT* mutation and more advanced disease.

Besides MC, eosinophils and basophils are those subsets of leukocytes that most frequently carry the *KIT* mutation in mastocytosis patients presenting with multilineage involvement of hematopoietic cells, in association with partial expression of CD117 in the later cells.^35^ Since both eosinophils and basophils might also be activated by NSAIDs and release leukotrienes due to PGE_2_ depletion,^41^ they could also play a role in these drug‐induced reactions in mastocytosis. Whether the *KITD816V* mutation has consequences on the activation threshold or the functionality of eosinophils and basophils in mastocytosis patients with mutilineage *KIT* mutation, and whether they are directly involved in DHRs to NSAIDs and other COXi, remains unknown and warrants further investigations, particularly among mastocytosis patients presenting with eosinophilia. Alternatively, a higher MC burden and/or specific alterations previously reported in the lipid membrane metabolism of MC from BMM versus advanced SM,^42^, ^43^ might also contribute to further explain the association here reported between DHR to NSAIDs or other COXi and (signs of) more advanced disease.

Similarly to the general population, propionic acid derivatives were the most frequent cause for NSAID hypersensitivity, which might be due to the greater use of these versus other NSAIDs, for all age groups.^40^ In our series however, these were amongst the safest NSAIDs, as only a small fraction (≤8%) of mastocytosis patients that had used ibuprofen, naproxen and dexketoprofen referred reactions to these drugs. In contrast, a significantly higher rate of hypersensitivity to ASA and metamizole was observed among patients who had received these drugs. Aceclofenac and clonixin were also associated with frequent reactions, but the number of patients who received them is rather limited to draw any definitive conclusions.

Previous studies suggested that mastocytosis patients who have hypersensitivity to ASA, would be tolerant to NSAIDs,^40^ which could only be confirmed here for a small number of single reactor patients (*n* = 3). Since ASA has a (slightly) preferential COX‐1 inhibitory action, it might be expected that it is frequently associated with hypersensitivity reactions to multiple different drugs, as found here. A previous study showed that ASA might be safe in 98% of mastocytosis patients with no history of NSAID DHR and that are able to tolerate being out of anti‐mediator treatment, as proven by drug challenge testing with ASA.^20^ This discrepancy may be explained by the fact that our data show that patients that most often react to NSAIDs or other COXi are the most symptomatic and more prone to anaphylaxis due to other causes. Despite of the (previously shown) remarkable rate of tolerance, performing drug challenge tests with the safest drug according to the characteristics of each patient (e.g., previous NSAIDs DHRs, baseline MCA symptoms, scheduled anti‐mediator therapy) may be a safer alternative, as anaphylaxis due to ASA seems to be common among patients with DHR to NSAIDs. In turn, metamizole and other pyrazolones are weak COX‐1 inhibitor analgesic drugs^44^, ^45^ that are commonly prescribed in Spain, and are reported to frequently cause IgE‐mediated reactions.^24^ This drug is not commonly prescribed in other countries and may explain regional discrepancies between the prevalence of NSAID or other COXi DHR found in ours versus other mastocytosis patient cohorts. In our series, pyrazolones were the most frequent elicitors of DHR among single reactors, but they were also frequent culprits among multiple reactors, suggesting a COX‐1 inhibition mechanism, as previously reported in the general population.^24^

In our study, reactions to paracetamol (a weak COX‐1 inhibitor) and coxibs (selective COX‐2 inhibitors) were found to be infrequent in adult mastocytosis patients and absent in children, in line with previous observations in non‐mastocytosis multiple reactors.^28^ Of note, tolerance to paracetamol, coxibs and/or to meloxicam was confirmed in the vast majority of multiple reactors, at higher frequencies than previously reported for patients with NSAID‐exacerbated respiratory disease.^23^ In fact, our results indicate that the rare reactions to paracetamol and preferential/selective COX‐2 inhibitors tend to occur only in multiple reactors, similarly to what has been reported for the general population.^14^

Mastocytosis patients who reacted against a NSAID or other COXi seem to be at greater risk of being multiple reactors. Moreover, mastocytosis patients may benefit from using these drugs although this deserves further investigation because a remarkably high percentage of both our adult and pediatric patients had never used NSAIDs after the onset of mastocytosis. Thus, our ultimate goal was to design an algorithm that could contribute to early identification of those mastocytosis patients who are at higher risk of being multiple reactors. Multivariate analysis showed that female sex, prior history of anaphylaxis not caused by NSAIDs, other COXi or HVA, presence of baseline flushing, sBT levels ≥48 ng/ml and multilineage *KITD816V* mutation were independent predictors for being a multiple reactor. In contrast, predictors for tolerance to NSAIDs and other COXi apart from paracetamol and hypersensitivity to only one NSAIDs or other COXi included: male sex, prior anaphylaxis due to HVA, and presence of (mastocytosis) skin lesions in the absence of baseline pruritus. Interestingly, the multiple reactor phenotype seems to oppose the BMM and HVA phenotype, that more frequently includes males, with no MCA‐associated symptoms other than anaphylaxis, low sBT and BMMC burden, in the absence of multilineage *KIT* mutation.^37^ Based on these results, a score model was built with a high sensitivity (associated with optimal negative predictive value), but a still limited positive predictive value, that would allow identification of one multiple reactor among each seven patients identified to be at risk.

**FIGURE 2:**
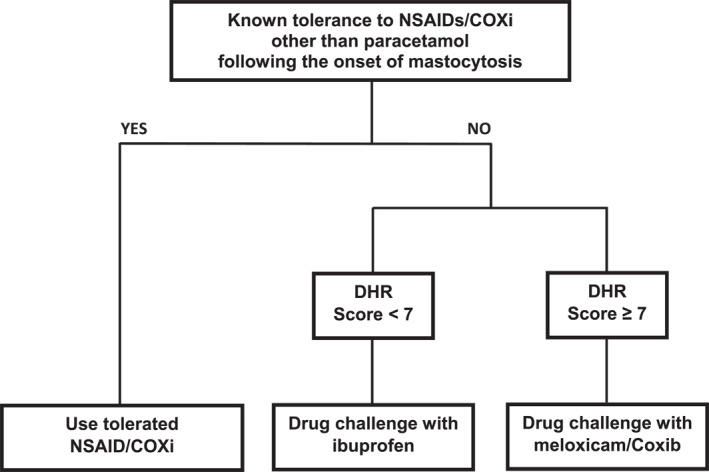
Decision‐tree algorithm for adult mastocytosis patients at risk of drug hypersensitivity reaction and associated with the administration of non‐steroidal anti‐inflammatory drugs and other cyclo‐oxygenase inhibitors apart from paracetamol

Based on these results, it might be wise that adult mastocytosis patients who require NSAIDs and who had never used them are submitted to a drug challenge with ibuprofen when the score here proposed is negative, or with either a coxib or meloxicam when the score is positive. In our experience, ibuprofen is safe in most children, but the first administration should be performed under medical surveillance whenever previous tolerance to the drug is not known (Figure 2). Patients who have tolerated specific NSAIDs following the onset of the disease do not require further testing and may be instructed to use the previously tolerated drug(s)^11^, ^46^ (Figure 2). Despite all the above, the score model here proposed has several limitations. The first relates to the fact that it was derived from a patient cohort studied in a reference mastocytosis center in which assessment of multilineage involvement of hematopoiesis by the *KIT* mutation is readily accessible. The second comes from being based on a still limited number of hypersensitive patients (20/332 for model 1 and 17/332 for model 2) in whom we could not systematically confirm that the identified NSAID or other COXi actually caused the (reported) DHR through diagnostic drug challenges, due to unfavorable risk/benefit analysis (high frequency of anaphylaxis) or refusal by patients. Instead, drug challenges with alternative drugs were carried out. While the first issue may be tackled by using the *KITD816V* mutation allele burden in PB as a surrogate marker for multilineage *KITD816V* mutation (which was not routinely performed in this series, at the time of diagnosis),^47^, ^48^ the second issue derives from hypersensitivity to NSAIDs or other COXi being an infrequent finding in patients with mastocytosis and warrants further prospective studies in larger (e.g., multicentric) series of patients in whom diagnostic drug challenges are performed.

In summary, our results show a higher frequency of MCA symptoms triggered by NSAIDs and other COXi in adult mastocytosis versus the general population, which are frequently associated with anaphylaxis and signs of more extensive disease, particularly among reactors to multiple drugs, in the absence of drug‐reaction‐associated deaths. Based on these results, mastocytosis should not be considered as a contraindication for the administration of NSAIDs or other COXi. However, it is strongly recommended that only drugs with a higher safety profile such as coxibs, meloxicam and paracetamol are used in adults with a positive score, while, in children and in adults with a negative score ibuprofen would be preferred. If tolerance to COX inhibitors is unknown, a controlled drug challenge with the aforementioned drugs is recommended, particularly among patients presenting with features of more advanced disease in the absence of HVA‐associated anaphylaxis and a positive score for hypersensitivity to multiple NSAIDs or other COXi drugs. Further studies in large series of adult and (particularly) pediatric patients are needed to validate these findings and recommendations.

## CLINICAL IMPLICATIONS

Hypersensitivity to NSAIDs and other COXi in mastocytosis patients is less frequent than previously estimated and it is associated with unique disease features.

## CONFLICT OF INTEREST

The authors have no conflicts of interest relevant to this article to disclose.

## AUTHOR CONTRIBUTIONS

**Tiago Azenha Rama:** Conceptualization; Data curation; Formal analysis; Investigation; Methodology; Writing – original draft; **José Mário Morgado:** Data curation; Formal analysis; Writing – review & editing ; **Ana Henriques:** Writing – review & editing; **Luis Escribano:** Writing – review & editing; **Iván Alvarez‐Twose:** Writing – review & editing; **Laura Sanchez Munoz:** Writing – review & editing; **Andre Moreira:** Writing – review & editing; **José Romão:** Writing – review & editing; **Alberto Orfao:** Supervision; Validation; Writing – review & editing; **Almudena Matito:** Data curation; Methodology; Project administration; Supervision; Validation; Writing – review & editing.

## Supporting information

FIGURE S1

FIGURE S2

TABLE S1

TABLE S2

## Data Availability

The data that support the findings of this study are available from the corresponding author upon reasonable request.
