# Supplementary material for: Mastocytosis presenting with mast cell‐mediator release‐associated symptoms elicited by cyclo oxygenase inhibitors: prevalence, clinical, and laboratory features
Source: Clin Transl Allergy. 2022 Mar 16;12(3):e12132. doi: 10.1002/clt2.12132 (PMC8967266; doi:10.1002/clt2.12132)
Supplement: Supplementary file 1 — FIGURE S1 [file CLT2-12-e12132-s002.docx]

**RETROSPECTIVE COHORT:**

**641 patients**

*(418 ≥ 18 y/o and 223 <18 y/o)*

**Excluded: n = 82**, *all <18 y/o*

*(insufficient/inconsistent data)*

**Enrolled: n = 559**

*(418 ≥ 18 y/o and 141 <18 y/o)*

**Included in the analysis:**

**n = 469**

*(382* ≥ 18 y/o *and 87* <18 y/o)

**Excluded: n = 90**

*(36 ≥ 18 y/o and 54 <18 y/o*

*who never received COXi other than paracetamol)*

**Supplementary Figure 1****.** **Flow chart illustrating how patients were selected or excluded from the study.** COXi, cyclo-oxygenase inhibitors; y/o, years old.
