# Supplementary material for: Mastocytosis presenting with mast cell‐mediator release‐associated symptoms elicited by cyclo oxygenase inhibitors: prevalence, clinical, and laboratory features
Source: Clin Transl Allergy. 2022 Mar 16;12(3):e12132. doi: 10.1002/clt2.12132 (PMC8967266; doi:10.1002/clt2.12132)
Supplement: Supplementary file 2 — FIGURE S2 [file CLT2-12-e12132-s004.docx]

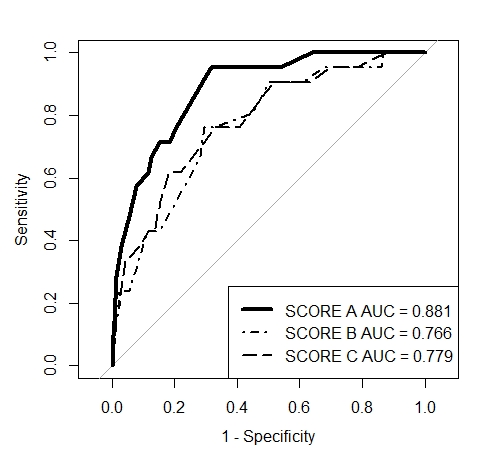


| **Score B** | **Score C** |
| --- | --- |

| Variable | Score |
| --- | --- |
| *Gender* |  |
| Female | +6 |
| Male | -2 |
| *Diagnosis* |  |
| Non-advanced mastocytosis | -5 |
| *Manifestations* |  |
| Flushing | +4 |
| Absense of pruritus | -3 |
| Skin lesions | -3 |
| Anaphylaxis caused by elicitors other than NSAIDs/COXi or hymenoptera venom | +5 |
| *Laboratory findings* |  |
| Multilineal D816V *KIT* mutation* | +4 |
| sBT ≥ 48 ng/mL | +6 |

| Variable | Score |
| --- | --- |
| *Gender* |  |
| Female | +6 |
| Male | -3 |
| *Diagnosis* |  |
| Non-advanced mastocytosis | -5 |
| *Manifestations* |  |
| Flushing | +4 |
| Absense of pruritus | -4 |
| Skin lesions | -3 |
| Anaphylaxis not caused by NSAIDs/COXi | +5 |
| Anaphylaxis caused by Hymenoptera venom | -6 |
| *Laboratory findings* |  |
| Multilineal D816V *KIT* mutation* | +4 |
| sBT ≥ 48 ng/mL | +5 |

**Supplementary Figure 2.**  **Receiver operating curves for Scores B and C as compared with Score A, for the screening of hypersensitivity to multiple NSAIDs and other COXi, in adult patients with mastocytosis.** AUC, area under the curve; COXi, Cyclooxygenase inhibitors; HVA, *Hymenoptera* venom allergy; MC, Mast cell; NSAIDs, nonsteroidal antiinflammatory drugs; sBT, serum baseline tryptase; SM, systemic mastocytosis. *If unknown, count as negative
