# Supplementary material for: Mastocytosis presenting with mast cell‐mediator release‐associated symptoms elicited by cyclo oxygenase inhibitors: prevalence, clinical, and laboratory features
Source: Clin Transl Allergy. 2022 Mar 16;12(3):e12132. doi: 10.1002/clt2.12132 (PMC8967266; doi:10.1002/clt2.12132)
Supplement: Supplementary file 3 — TABLE S1 [file CLT2-12-e12132-s003.doc]

|  | |  | **CM** | | **MIS** | | **ISM** | | **BMM** | | **SSM** | | **ASM** | | **ISM-AHN** | | **MCL** | | ***p*** | |
| --- | --- | --- | --- | --- | --- | --- | --- | --- | --- | --- | --- | --- | --- | --- | --- | --- | --- | --- | --- | --- |
| **Adults** | | |  | |  | |  | |  | |  | |  | |  | |  | |  | |
|  | | *HVA* | 1  (20%) | | 1 (100%) | | 10  (19%) | | 57  (53%) | | 0  (0%) | | 1  (17%) | | 0  (0%) | | 0  (0%) | | <0.001 | |
|  |  | *Drug allergy* | 2  (40%) | | 0  (0%) | | 26  (48%) | | 15  (14%) | | 1 (100%) | | 5  (83%) | | 0  (0%) | | 0  (0%) | | <0.001 | |
|  |  | *Idiopathic* | 2  (40%) | | 0  (0%) | | 11  (20%) | | 9  (8%) | | 0  (0%) | | 0  (0%) | | 0  (0%) | | 0  (0%) | | NS | |
|  |  | *Mixed* | 0  (0%) | | 0  (0%) | | 2  (4%) | | 14  (13%) | | 0  (0%) | | 0  (0%) | | 0  (0%) | | 0  (0%) | | NS | |
|  |  | *Food allergy* | 0  (0%) | | 0  (0%) | | 5  (9%) | | 11  (10%) | | 0  (0%) | | 0  (0%) | | 0  (0%) | | 0  (0%) | | NS | |
|  |  | *Other insects* | 0  (0%) | | 0  (0%) | | 0  (0%) | | 1  (1%) | | 0  (0%) | | 0  (0%) | | 0  (0%) | | 0  (0%) | | NS | |
|  | | **Total** | **5/22 (23%)** | | **1/24**  **(4%)** | | **54/197**  **(27%)** | | **107/115**  **(93%)** | | **1/4**  **(25%)** | | **6/10 (60%)** | | **0/7**  **(0%)** | | **0/3 (0%)** | | **<0.001** | |
| **Children and adolescents** | | | | |  | |  | | - | | - | | - | | - | | - | |  | |
|  | | *Drug allergy* | 2  (67%) | | - | | 1  (100%) | | - | | - | | - | | - | | - | | NS | |
|  |  | *Idiopathic* | | 1  (33%) | |  | | 0  (100%) | | - | | - | | - | | - | | - | | NS |
|  | **Total** | | **3/86**  **(4%)** | | **-** | | **1/1 (100%)** | | **-** | | **-** | | **-** | | **-** | | **-** | | **<0.001** | |

**Supplementary Table I. Causes of anaphylaxis distributed according to the diagnostic subtype of mastocytosis.**

Results expressed as number of patients with specific causes for anaphylaxis for each diagnostic subtype of mastocytosis and percentage between brackets. ASM, aggressive systemic mastocytosis; BMM, bone marrow mastocytosis; CM, cutaneous mastocytosis; ISM, indolent systemic mastocytosis; ISM-AHN, indolent systemic mastocytosis with an associated hematological neoplasm MC, mast cells; MCL, mast cell leukemia; MIS, mastocytosis in the skin; NS, not statistically significant; SSM, smouldering systemic mastocytosis.
