# Supplementary material for: Mastocytosis presenting with mast cell‐mediator release‐associated symptoms elicited by cyclo oxygenase inhibitors: prevalence, clinical, and laboratory features
Source: Clin Transl Allergy. 2022 Mar 16;12(3):e12132. doi: 10.1002/clt2.12132 (PMC8967266; doi:10.1002/clt2.12132)
Supplement: Supplementary file 4 — TABLE S2 [file CLT2-12-e12132-s001.doc]

**Supplementary Table II. Demographic, clinical and laboratory characteristics of pediatric mastocytosis patients grouped according to their tolerance profile to NSAIDs and other COX inhibitor drugs (n = 87).**

|  | | **NSAID tolerants**  **(n=79)** | **Hypersensitivity to NSAIDs**  **(n=8)** | ***p*** |
| --- | --- | --- | --- | --- |
| **Sex (female)** | | 34 (42%) | 4 (50%) | *NS* |
| **Age** | | 10 (3-17) | 11 (5-17) | *NS* |
| **Age at onset (years)** | | 0 (0-7) | 0 (0-8) | *NS* |
| **Diagnosis** | MPCM | 65 (83%) | 6 (75%) | *NS* |
|  | Cutaneous mastocytoma | 9 (11%) | 1 (13%) | *NS* |
|  | DCM | 2 (3%) | 1 (13%) | *NS* |
|  | WDSM | 3 (4%) | 0 (0%) | *NS* |
| **Basal MC mediator release related symptoms** | Flushing | 27 (34%) | 3 (38%) | *NS* |
|  | Pruritus | 42 (53%) | 6 (75%) | *NS* |
|  | GI symptoms | 23 (30%) | 2 (25%) | *NS* |
|  | Anaphylaxis | 3 (4%) | 1 (13%) | *NS* |
| **MC mediator release related symptoms during DHRs** | Pruritus | - | 3 (38%) | - |
|  | Urticaria | - | 4 (50%) | - |
|  | Angioedema | - | 1 (13%) | - |
|  | Vomiting | - | 2 (25%) | - |
|  | Diarrhea | - | 2 (25%) | - |
|  | Flushing | - | 2 (25%) | - |
|  | Anaphylaxis | - | 2 (25%) | **-** |
| **Allergic sensitization** | | 27 (34%) | 2 (25%) | *NS* |
| **Allergic diseases** | Rhinoconjunctivitis | 20 (25%) | 1 (13%) | *NS* |
|  | Asthma | 9 (11%) | 1 (13%) | *NS* |
|  | Atopic dermatitis | 10 (13%) | 1 (13%) | *NS* |
|  | Food allergy | 5 (6%) | 2 (25%) | *NS* |
|  | HVA | 1 (5%) | 0 (0%) | *NS* |
| **Laboratory Findings** | IgE (kU/L) * | 33.1 (2-669) | 46.9 (28-116) | *NS* |
|  | Eosinophils absolute count (x10^9^/L)** | 0.225 (0.054-5.98) | 0.3 (0.07-0.5) | *NS* |
|  | sBT (ng/mL) | 5.6 (1.1-68.7) | 9.39 (1.1-149) | *NS* |
| **Follow-up (years)** |  | 10 (3-17) | 9 (5-12) | *NS* |

Results expressed as number of patients and percentage between brackets (rounded to units) or as median values and range between brackets. *Analyzed in 62 patients; ** Studied in 75 patients **

BM: bone marrow; HVA, *Hymenoptera* venom anaphylaxis; NS: not statistically significant; NSAIDI: Intolerants to nonsteroidal anti-inflammatory drugs; MC: mast cells; sBT: serum baseline tryptase;
